# A matched-filter technique with an objective threshold

**DOI:** 10.1038/s41598-022-25839-2

**Published:** 2022-12-21

**Authors:** Shiro Hirano, Hironori Kawakata, Issei Doi

**Affiliations:** 1 Department of Physical Science, College of Science and Engineering, Ritsumeikan University, 1-1-1, Nojihigashi, Kusatsu, Shiga 525-8577 Japan; 2 Disaster Prevention Research Institute, Kyoto University, Gokasho, Uji, Kyoto 611-0011 Japan

**Keywords:** Geophysics, Seismology

## Abstract

We propose an objective threshold determination method for detecting outliers from the empirical distribution of cross-correlation coefficients among seismic waveforms. This method is aimed at detecting seismic signals from continuous waveform records. In our framework, detectability is automatically determined from Akaike’s Information Criterion (AIC). We applied the method of seismic signal detection to continuous records collected over two years. The results show that the maximum value of network cross-correlation coefficients sampled from each constant interval can be approximated by the theory of extreme value statistics, which provides a parametric probability density function of maxima. By using the function, outliers can be considered with a reasonable criterion.

## Introduction

A matched-filter (MF) analysis, a technique for quantifying the similarity between continuous and template waveforms using the cross-correlation coefficient (CC), is efficient in detecting weak seismic signals embedded in continuous waveform records^1^. Many types of seismic events have been detected automatically using MF analysis: non-volcanic tremors and low-frequency earthquakes^2–4^, seismic swarms^5,6^, foreshocks, and aftershocks^7–10^. In general MF analyses, waveforms are regarded as seismic signals when the CC between a template and continuous waveform exceeds a threshold value. The threshold value has occasionally been defined as a constant^9,10^ or not specified^7^. However, given the possibility of relatively high CC values randomly occurring for microtremors, the threshold should be determined depending on the empirical frequency distributions of CC. In other previous studies, the threshold value was defined as a constant factor multiplied by the standard deviation ($\sigma$)^3,4^ or the median absolute deviation (MAD)^2^. Under this strategy, we can estimate the possibility of a false positive if a probability density function (PDF) of the CC is known. Thus, the characteristics of the PDF should be investigated both theoretically and experimentally. Because event detection is a type of outlier detection, careful attention should be given to the tails of the frequency distribution of CC; do they follow the Gaussian, exponential, or power-law? In some cases^2–4^, the Gaussian distribution has been assumed, and the possibility of false positives was calculated under this assumption. However, to plot a Gaussian curve overlapping the histogram of CC in a linear scale is insufficient because their tails are invisibly small compared to the peak, and a plot in a semi-log scale is required. In this manner, only Aso et al.^4^ showed that the tails follow the Gaussian. In this study, we first derive a normal distribution that the CC between random microtremor and random template waveform may follow and investigate the effect of a band-pass filter, which provides a reference for determining a realistic CC distribution. In this context, we reveal a statistical background and nature of the conventional MF analysis. Next, we consider a distribution that the maximum value of CC in every constant interval follows for robust outlier detection using non-random continuous waveform records. The distribution of maxima in every constant interval is given by the extreme value theory^11^. Subsequently, we demonstrate that the tails of CC values are well modelled by the extreme value distribution rather than the normal distribution through a case study of 2-years continuous records and multiple templates of foreshocks before an M5.4 crustal earthquake in Japan. Such modelling was also done^12^ for long-term waveform data recorded by ocean-bottom seismometers. However, they^12^ considered the top 5% of CC values significant seismic signals even though the values obeyed the extreme value distribution. We have to note that almost all CC values are due to background noises rather than rare seismic signals, and significant signals should be outliers deviating from the background distribution due to the noise. Given the extreme value distribution, we develop a reasonable method for detecting outliers based on Akaike’s Information Criterion (AIC). Moreover, we provide a case study of event detection. We focus on a specific foreshock activity and should conduct more verification with various data sets. However, the method proposed in this study would be applicable in principle for other seismic phenomena and regions where CC between a template and continuous long-term waveforms are available. In summary, the aims of this study are as follows: (1) to clarify the theoretical background and limitation of a conventional MF analysis with a subjective threshold, and (2) to propose a new method to determine an objective threshold based on theories of the extreme value statistics and Akaike’s information criterion.

## Theory and method

### Ideal frequency distribution of CC

**Figure 1:**
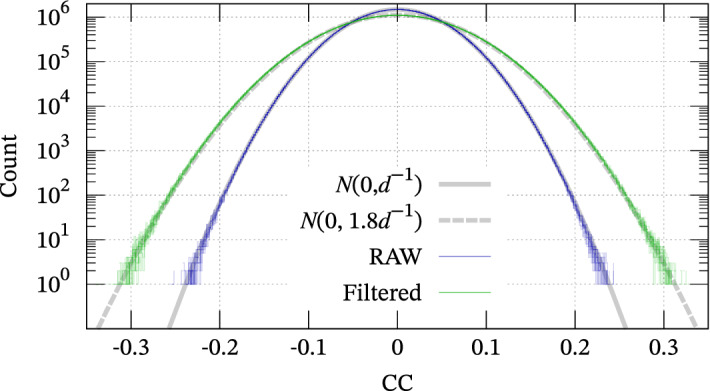
Frequency distribution of CC in a numerical experiment. CC between a raw random noise and a random template follows the normal distribution $N\left( 0, d^{-1}\right)$, whereas CC between a filtered random noise and the random template follows $N\left( 0, 1.8 \, d^{-1}\right)$.

In the following, without loss of generality, we regard velocity seismograms as the data. The frequency distribution of CC between a continuous record and an arbitrary template waveform array of length $d$ follows a normal distribution whose mean is zero and variance is $d^{-1}$ if the continuous record is a time series of independent and identically distributed (i.i.d.) random variable. Let $d$-dimension vectors $\varvec{u} := (u_i)$ and ${\varvec{v}} := (v_i)$ $(i=1,2,\ldots ,d)$ be discretized and offset-eliminated waveform arrays of length $d$. Their CC is given as

1 $$\begin{aligned} CC = {\widehat{\varvec{u}}} \cdot {\widehat{{\varvec{v}}}}, \end{aligned}$$

where ${\widehat{\varvec{u}}}$ and ${\widehat{{\varvec{v}}}}$ are normalized $\varvec{u}$ and ${\varvec{v}}$, respectively, by their $L^2$-norm. If ${\varvec{v}}$ is extracted from a random waveform, the normalized vector ${\widehat{{\varvec{v}}}}$ is an isotropic random vector restricted on the ($d-1$)-dimensional unit sphere. Because Eq. (1) is a projection of ${\widehat{{\varvec{v}}}}$ along the ${\widehat{\varvec{u}}}$-direction, CC can be regarded as a velocity component along the ${\widehat{\varvec{u}}}$-direction of randomly hurtling particles with unit velocity $\left( \left| {\widehat{{\varvec{v}}}} \right| \equiv 1 \right)$. Therefore, the PDF of CC can be approximated by extending the Maxwell-Boltzmann distribution from 3-dimensional to $d$-dimensional space; see also Supplement A. In fact, the template and continuous waveform are filtered in MF analyses because seismic waveforms have high S/N ratios in some limited frequency bands. Linear band-passed filtering is equivalent to the convolution of a characteristic function and the continuous waveform. Therefore, ${\varvec{v}}$ is not an i.i.d. random vector but necessarily has dependence among some neighbour samples (referred to as “coherence”) depending on the filter. Thus, we conducted numerical experiments; we calculated CC between an i.i.d. random waveform of length $10^8$ and a random array of length $d = 500$ (Fig. 1). After ten experiments, we confirmed that CC follows the normal distribution $N\left( 0, d^{-1}\right)$ as expected above and in Supplement A. On the other hand, if we regard the waveforms as 100 Hz time-series and apply a band-pass filter of 5–30 Hz that is required in the next section, we find that the distribution is approximated as $N\left( 0, 1.8 \, d^{-1}\right)$, as shown in Fig. 1, because narrowing the frequency band may increase the variance; ultimately, monochromatic signals yield high correlation frequently. Therefore, we can conclude that CC follows the normal distribution even after applying the band-pass filter if the continuous waveform is random.

### Frequency distribution of the maximum of CC

The assumption of i.i.d. in the previous subsection might not be valid in cases where multiple similar earthquake events frequently occur, radiating waveforms similar to the templates, or the microtremor repeats similar patterns. Indeed, power spectral densities of ambient noise imply that they have some correlation^13,14^. In such cases, even accidentally, relatively high CC values appear around their local peaks because of the coherence. Hence, the frequency distribution of all CC values will be contaminated by the high values repeatedly, thus rendering the tail of the distribution wider and the interpretation more difficult. To avoid this problem, outliers should be detected from the maximum value of CC in every $M$ sample by assuming that the coherence of microtremors or seismic waveforms is lost within $M$ samples. This assumption is valid because, generally, shorter-term correlation is stronger than longer-term correlation. After picking the maxima in every $M$-sample interval, we need a model to describe a PDF that the maxima follow. The extreme value statistics provide the PDF and enable us to extrapolate an empirical distribution of the maxima and predict the possibility that the maxima will become extremely large. The extreme value statistics are often contrasted with the central limit theorem; the former and the latter describe the PDF of the maxima and the mean, respectively, of an i.i.d. random variable generated by any distribution. Theoretically, it has been shown that the frequency of the maxima of any distribution in every interval follows the Generalized Extreme Value (GEV) distribution^11,15^, which has been employed to model possibilities of rare events, such as floods and economic crises. GEV has the following cumulative density function (CDF):

2 $$\begin{aligned} F_{\text {GEV}}\left( x \mid \mu ', \sigma ', k\right) = \exp \left( -\left( 1 + k \frac{(x-\mu ')}{\sigma '} \right) ^{-1/k} \right) , \end{aligned}$$

where $x$ is a random variable, and $\mu ', \sigma ',$ and $k$ are the location parameter, scale parameter, and shape parameter, respectively. We have to note that ${\text {sgn}}(k) \left( x-\mu '+\sigma '/k\right) \ge 0$ must be satisfied; otherwise, the PDF is zero therein. It may be possible to detect outliers by fitting the distribution of the maxima with GEV even if CC does not follow the normal distribution while their maxima follow GEV; see Supplement B for the maximum likelihood estimation (MLE) of the GEV parameters. In particular, if every interval contains sufficient data, the cumulative distribution converges to one of three specific cases depending on the shape of their tail: the Gumbel distribution, Fréchet distribution, or Weibull distribution. In the next section, we assume that the CDF of the Gumbel distribution, $F_{\text {G}}$, can approximate them:

3 $$\begin{aligned} \begin{aligned} F_{\text {G}}\left( x \mid \mu ', \sigma '\right)&: \,= \lim _{k \rightarrow 0} F_{\text {GEV}}\left( x \mid \mu ', \sigma ', k\right) \\&= \exp \left( -\exp \left( -\frac{x - \mu '}{\sigma '}\right) \right) . \end{aligned} \end{aligned}$$

This is because of the following reasons: (1) as confirmed in the next section, the accumulated data distribution shows linear falloff in semi-log plots, which is a characteristic of the Gumbel distribution, and (2) as in Supplement B, MLE of three parameters for GEV is technically challenging in some case. We focus on and plot $1 - F_{\text {G}}$ in the following.

### Method for event detection

Although the threshold for MF analyses has widely been assumed from the data histogram, we have no unified or objective algorithm to assume an appropriate threshold value. Here, we propose an algorithm for detecting outliers reasonably and objectively based on an information criterion. Akaike’s Information Criterion (AIC) provides the most likely number of parameters of a model PDF so that the Kullback–Leibler divergence (or relative entropy) between the model PDF and the true (but unknown) PDF is minimized^16^. According to AIC, the number of parameters that minimizes AIC (= − 2$\log$-likelihood + 2 $\times$ number of parameters) is the most likely. The elimination of outliers for minimizing AIC has been developed in applied statistics^17–20^ and implemented in bioinformatics^21^, assuming that the outliers have exceptionally large values. In this context, “the number of parameters” is “the number of outliers + the number of parameters for a model PDF that data other than the outliers follow”. The pioneers^17–19,21^ assumed that the random variable other than the outliers followed a normal distribution and calculated AIC; Marmolejo-Ramos et al.^20^ investigated the applicability of the method in non-Gaussian and skewed distribution cases. We assume the Gumbel distribution and calculate the difference in AIC when we increase the number of suspects, indicating whether the increment of the number is reasonable.

We sort $N$ data into the descending order ($x_1> x_2> \cdots > x_N$) and assume that the leading $s$ data ($x_1, x_2, \ldots , x_s$) are outliers that do not follow the Gumbel distribution while the other $N-s$ data are sampled from the same Gumbel distribution. Note that, unlike our notation, Ueda^18,19^ sampled $N$ data out of $N+s$ data. Then, AIC with the $s$ outliers is represented as^18–20^

4 $$\begin{aligned} \frac{1}{2} \text {AIC}_s = - \sum _{j=s+1}^N \log {f(x_j \mid \theta ')} - \log {(N-s)!} + s , \end{aligned}$$

where $f$ is the assumed PDF the samples follow, and $\theta '$ is the maximum likelihood parameter. In the original method, $f$ has been assumed to be the normal distribution^18,19^. However, the original method tends to be sensitive and detect too many values as outliers if the true distribution is positively skewed^20^. In our case, we assume that the true distribution is approximated by the Gumbel distribution, which has positive skewness. Therefore, instead of the normal distribution, $f(x_j \mid \theta ) = p_G(x_j \mid \mu ', \sigma ')$ should be considered, where $p_{\text {G}} :\,= \frac{d F_{\text {G}}}{d x}$ is the PDF of the Gumbel distribution.

In the following, we do not directly calculate Eq. (4) that contains an uncalculatable huge number $\log {(N-s)!}$ for our case ($N \sim 10^6$). Instead, for sufficiently large $N$, the difference in AIC between the cases of $s$ outliers and $s+1$ outliers, $\frac{1}{2} d\text {AIC}_s$, can be approximated as

5 $$\begin{aligned} \begin{aligned} \frac{1}{2} d\text {AIC}_s :\, =&\frac{1}{2} \left( \text {AIC}_{s+1} - \text {AIC}_s\right) \\ \sim&\log {p_{\text {G}}\left( x_{s+1} \mid \mu ', \sigma ' \right) } + \log {(N-s)} + 1. \end{aligned} \end{aligned}$$

Strictly, the maximum likelihood parameters based on all $N$ data could differ from those estimated using $N-s$ or $N-s-1$ data. However, we assume that $N \gg s$ holds and the parameters do not change significantly after the elimination of $s$ data; see also Supplement C on its effect. Because we focus on the right tail of $p_{\text {G}}$ and $x_i$ is in descending order, $p_{\text {G}}(x_s) < p_{\text {G}}(x_{s+1})$ holds, which results in

6 $$\begin{aligned} \frac{1}{2} d\text {AIC}_s < \frac{1}{2} d\text {AIC}_{s+1}. \end{aligned}$$

**Figure 2:**
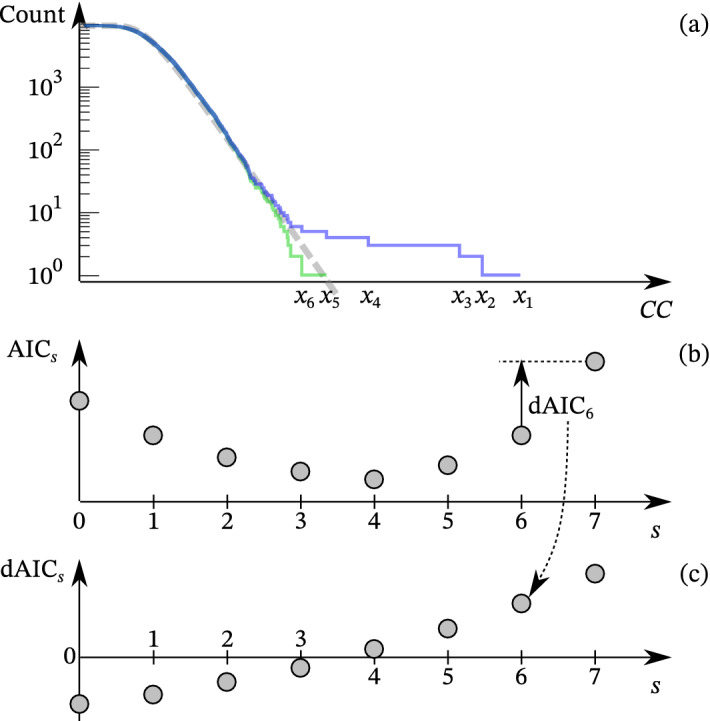
Schematic illustration for estimating $s_0 = 4$, where $s_0$ is the number of outliers out of $N = 10^4$. (**a**) Cumulative number of raw data (blue steps), estimated Gumbel distribution (gray broken line), and cumulative number of data after elimination of $x_1, \ldots , x_{s_0}$ (green steps). (**b**) Dependence of AIC on the number of outlier candidates, $s$. (**c**) Dependence of $d\text {AIC}_s := \text {AIC}_{s+1} - \text {AIC}_s$ on $s$, where the definition is exemplified for $s = 6$. Even though the blue step due to $x_5$ is above the gray line in (**a**), $x_5$ is not regarded as an outlier because the step becomes closer to the gray line after the elimination.

In other words, the difference in AIC is a monotonically increasing sequence. If $d\text {AIC}_s < 0$ holds, from the definition, we can reasonably regard that $x_{s+1}$ is also an outlier rather than a sample from the Gumbel distribution. On the contrary, if $d\text {AIC}_s > 0$ holds, the monotonicity guarantees that the difference is always positive as $s$ increases. Thus, all $x_i$ ($i > s$) are not outliers. Finally, our procedure schematically illustrated in Fig. 2 is as follows. We first obtain the MLE of the parameters $\mu '$ and $\sigma '$, then calculate $d\text {AIC}_s$ for $s = 0, 1, 2, \ldots$. We stop the calculation when $s$ reaches $s_0$, which makes $d\text {AIC}_s$ positive for the first time, and finally conclude that $x_1, x_2, \ldots , x_{s_0}$ are outliers.

**Table 1:** Detected events by the proposed algorithm.

ID	Date	Time	Similar to
A	2011-05-04	19:17:00	05, 06
B	2011-06-26	11:57:47	01, 02, 04, 14, 18, 27
C	2011-06-26	12:57:45	01, 02, 04, 18, 23, 27
D	2011-06-27	07:24:14	01, 02, 04, 18, 20, 23, 27

**Figure 3:**
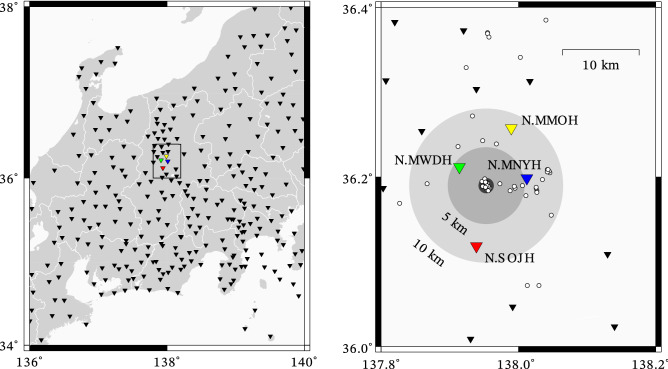
Distribution of observation points (triangles) and foreshock hypocentres (white circles) prior to an M5.4 mainshock in Nagano, Japan. Waveforms observed at N.MWDH (green), N.MNYH (blue), N.MMOH (yellow), and N.SOJH (red) stations were analyzed in this study. See Table S.1 for detail of the 27 events within 1 km from the epicenter shown in the darkest circle.

**Figure 4:**
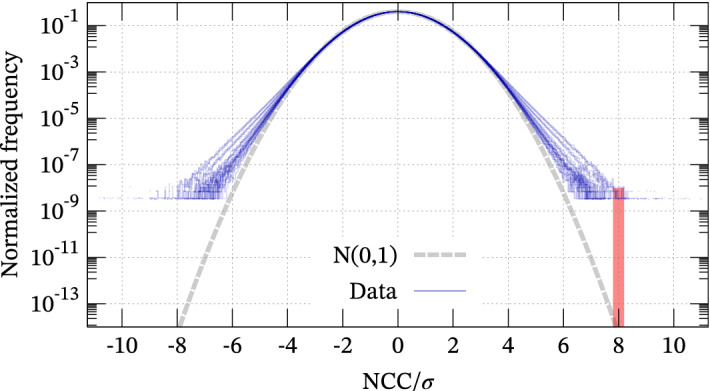
Empirical distribution of NCC between 2-year continuous records and 27 template waveforms (blue). The abscissa is normalized by the standard deviation. The vertical red bar indicates that the empirical distribution is several orders larger than the normal distribution (gray) at $8 \sigma$.

**Figure 5:**
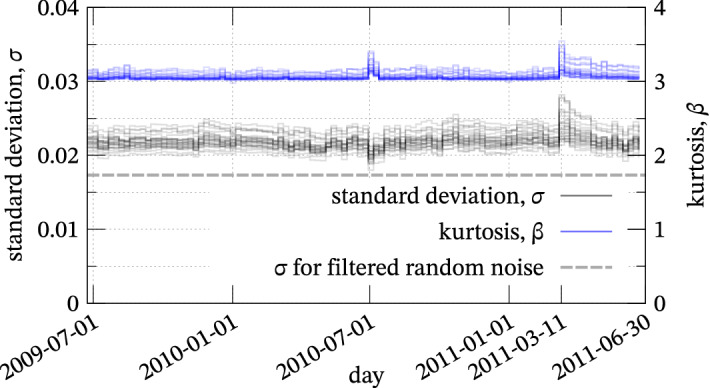
Temporal variation of the standard deviation and kurtosis of the empirical distribution of NCC.

**Figure 6:**
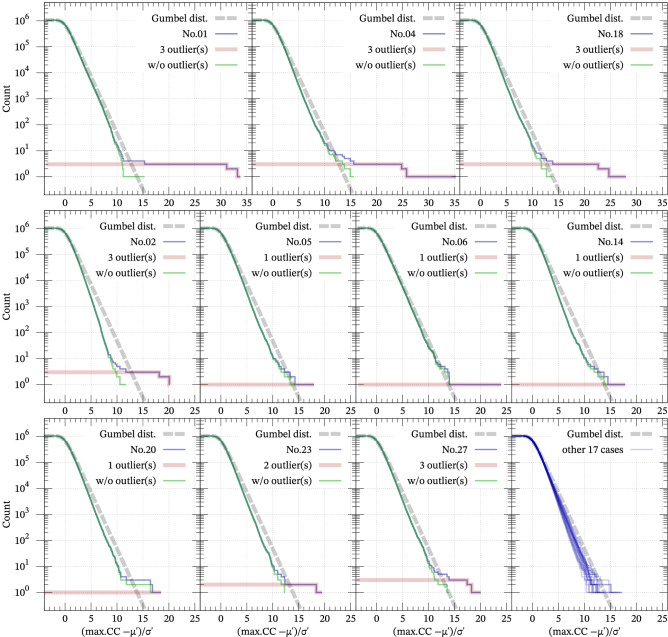
Cumulative distributions of normalized NCC (blue) and the Gumbel distribution with $\mu ' = 0$ and $\sigma ' = 1$ as the fitting curve (gray) for the 27 templates of Table S.1. Red steps indicate detected outliers in terms of the minimization of AIC. Seventeen cases accompanied by no outliers are plotted all together in the right bottom.

## Case study

### Data

We considered a foreshock sequence of an M5.4 earthquake: origin time = 2011-06-30 08:16:37.06 (Japan Standard Time); epicenter = 35.188${}^\circ$ N, 137.955${}^\circ$ E; depth = 4.3 km. According to the JMA catalog, 27 small foreshocks were recorded within 13 h before the mainshock (Table S.1); their epicenters are within 1 km from the epicenter of the mainshock and surrounded by four Hi-net observation stations within 10 km (Fig. 3), which may enable us to investigate the seismicity precisely. Thus, for each station and component, the 27 template waveforms were extracted from 0.5 s before each arrival of the P-wave, and their length was 5 s (= 500 samples), such that the significant part of the S-wave and its coda is included. To search for events similar to these foreshocks, a criterion for outlier detection based on the empirical distribution of CC is required. We thus calculated the Network Cross-correlation Coefficient (NCC) among template waveforms due to the 27 events and 2-year continuous waveforms between 2009-06-29 and 2011-06-28 before the activation of the foreshocks. NCC is the averaged value of CC obtained in each station and component after shifting CC by lags between the origin time and arrival time of P-wave^1^. Even after averaging, maxima of NCC should follow GEV because maxima generated by an arbitrary distribution follow GEV^11,15^. In our case, we stack 12 CC time series based on three components of the four stations and obtain 27 histograms of NCC in total. Before the calculation, we applied a band-pass filter to focus on the frequency band, in which waveforms due to foreshocks show high S/N ratios. Although Doi & Kawakata^9^ applied a band-pass filter of 15–40 Hz, we applied a filter of 5–30 Hz depending on the spectra of template waveforms; some automatic and objective determination methods of the band should be developed in the future. Hi-net waveforms include signals for checking the state of health of the observation instruments^22^. Thus, we eliminated 15-s daily data between 09:00:00.00 and 09:00:15.00 to ensure that the signals did not affect CC.

### Result: histogram of NCC

All histograms of NCC are shown in Fig. 4. The histograms were normalized by their standard deviation, meaning they should be well approximated by the standard normal distribution plotted by the gray parabola in the semi-log plot if NCC follows a normal distribution. However, the tails of the NCC histograms appear to be linear in the semi-log plot and significantly different from the theoretical distribution discussed in the previous section. The difference between the theoretical model and the empirical data is over a hundredfold at $7 \sigma$ and ten thousandfold at $8 \sigma$. This fact strongly implies that the observed microtremor is significantly far from the i.i.d. assumed in the ideal case and has non-negligible coherence. Weekly statistics of NCC histograms (Fig. 5) show that the standard deviation is higher than the case of the random waveform ($\sigma = \sqrt{1.8 \, d^{-1}}$, where $d = 500 \times 12$ in this case), which implies that the microtremor is somehow biased. Therefore, the possibility of false positives may be severely underestimated if we set the threshold value as $8 \sigma$^4,8^ and implicitly assume that the histogram follows a normal distribution. When the continuous waveform has coherence, relatively larger CC values tend to appear near their peaks because waveforms $u(t)$ and $v(t \pm \delta t)$ with sufficiently small $\delta t$ are also similar if $u(t)$ and $v(t)$ are quite similar waveforms. This means that we cannot count numbers of similar waveforms on the basis of Fig. 4 because higher CC values contain almost the same waveform (e.g., $v(t)$ and $v(t \pm \delta t)$). Hence, to eliminate such waveforms within the short term, we should refer to the distribution of the maxima of NCC that is less sensitive to coherence.

Figure 5 also shows that characteristics of histograms, such as the standard deviation and kurtosis, fluctuated immediately after the week, including those on March 11, 2011, the day the M9.0 Tohoku earthquake occurred. However, the two years were not separated in our analysis because a sufficient amount of data is required to investigate the tails of histograms.

### Result: cumulative distribution of max. of NCC

We attempted to detect seismic events that possibly occurred in the two years using the proposal in the “Theory and method” section after fitting the cumulative number of the maxima of NCC at every minute between 2009-06-29 and 2011-06-28. In total, we could select 21 outliers according to Fig. 6, which shows the cumulative number of calculated NCC, the estimated Gumbel distribution $1 - F_{\text {G}}\left( x \mid \mu ', \sigma '\right)$, and detected outliers. However, we classified some of these outliers as the same event because they emerged within 1 s. Finally, we could detect four new events (i.e., all the 21 outliers were from multiple counts of the four uncataloged events), as shown in Table 1, which the JMA has not cataloged. As shown in Figs. S.1–S.4, the detected waveforms show amplitudes of a maximum 10–20% of the template waveforms and, therefore, have relatively low S/N ratios compared to the template. Our method provided the seismic signals without any prescribed threshold, even from such noisy data. The finding of the triplet similar events 3–4 days before the mainshock in the foreshock region (IDs B–D in Table 1) may provide us with new insight for considering the preparation process of the mainshock.

## Discussion

Compared to conventional thresholding methods, the most important advantage of our method is that the results are objective and reasonable; the result is less affected by arbitrariness in principle. We can suggest the possibility of false positives under the Gumbel distribution because the differences between the distribution and the cumulative number of data are almost less than tenfold (Fig. 6). The conventional method involves a trade-off between the number of detected events and false positives depending on the threshold value. In our method, however, the detection criterion is automatically determined depending only on the data quality. Thus, our method provides an objective reference of the threshold value. If we lower the threshold below the reference, newly picked CC values are probabilistically from the same trends as the background noise because our method picked only outliers, and the remaining CC values are regarded as those embedded in the PDF due to the noise. We have to note that the above discussion is just probabilistic, and CC values lower than the reference threshold may be meaningful in some cases. For example, as in Fig. 6, the CC based on the template No.01 suggested three outliers, and the fourth largest CC value was rejected. However, the fourth-largest value corresponds to the event ID = A (Table 1). Similarly, the rejected third-, fifth-, and sixth-largest CC for the template No.06 correspond to ID = C, B, and D, respectively; we can see how they are (or are not) similar to each other in Figs. S.1–S.4 (e.g., “A” in Fig. S.1 and “01” in Figs. S.2–S.4). In such a case, we may consider that the similarity among them is not significant in terms of the information criterion, even though the waveforms are not noise.

As many seismologists wish, the lower threshold reduces the possibility of false negatives. Therefore, one may calculate the threshold value by our method and consider lowering it depending on one’s aim. Only four events were detected in our analysis, which may mean that the hypocentre region had been quite inactive before the foreshock activity or our method is excessively strict at finding many uncataloged events. Nevertheless, even if the latter is true, the detection of four uncataloged events shows that our method has a higher detection ability than JMA in that term, at least for similar seismic events.

It is noteworthy that our method is not completely free of arbitrariness. One concern is the length of intervals using which we selected the maxima. In our experiment, we selected an interval of 1 min (i.e., 6000 samples) considering computational time, but in principle, the interval can be, for example, 5 s (i.e., 500 samples). With longer lengths, the data distribution may converge to the GEV theoretically^11,15^. Still, the temporal resolution will decrease because relatively more minor peaks of CC values will be neglected if a higher peak emerges in the same interval, which becomes likely for longer intervals. In contrast, with shorter lengths, convergence might not be achieved. Therefore, we should determine the interval length considering the balance between the above two. Another concern is daily variation^4^ or long-term variation of CC as shown in Fig. 5. For precise analysis, the threshold should be determined in each short term (e.g., diurnal and nocturnal or seasonal distributions of CC). In such a case, our method can be applied to each term separately, although we ignored such variations for simplicity and because of the absence of an objective method to separate each term. For such short-term records, in general, the estimated parameters might have more errors due to the decrease in the sample size, which may limit the applicability of the proposed method. Therefore, quantifying the errors as in Supplement C is required for stable estimation of parameters depending on the short-term data.

Because our case study is limited, it remains unclear whether the Gumbel distribution can model the empirical distribution in general cases. Even in our results, the deviation of empirical distributions from the Gumbel distribution (Fig. 6) may imply that the Gumbel distribution is not necessarily the best model. A suitable approximation is possible using other limits of GEV: the Fréchet or Weibull distribution. In practice, the shape of the tail should be further investigated, considering these possibilities in each analysis. However, we face a technical problem in estimating three GEV parameters with outliers as in Supplement B, and the more suitable approximation remains for future work.

## Conclusion

We developed an objective matched-filter technique based on AIC and the extreme value theory. We showed that the CC between any template and i.i.d. random waveform follows the normal distribution, which provides a reference for examining the deviation of data from the i.i.d. case. To reduce the possibility of a false positive, we considered the maximum of CC in each interval and found that the maxima follow the Gumbel distribution. Finally, using the distribution and AIC, we propose a reasonable method less sensitive to arbitrariness than a conventional thresholding method for detecting outlier seismic signals. Regardless of whether NCC follows the normal distribution, the proposed method can be applied to analyses of seismic event detection.

## Supplementary Information

Supplementary Information.

## Data Availability

We used continuous waveform records of the NIED high sensitivity seismograph network of Japan ( https://doi.org/10.17598/NIED.0003 ) and JMA unified earthquake catalog ( https://www.hinet.bosai.go.jp/REGS/JMA/jmalist.php?LANG=en ).
