# Supplementary material for: A matched-filter technique with an objective threshold
Source: Sci Rep. 2022 Dec 21;12:22090. doi: 10.1038/s41598-022-25839-2 (PMC9772383; doi:10.1038/s41598-022-25839-2)
Supplement: Supplementary file 1 — Supplementary Information. [file 41598_2022_25839_MOESM1_ESM.pdf]

Supporting Information for

A matched-filter technique with an objective threshold

Shiro Hirano[1,*], Hironori Kawakata[1], and Issei Doi[2]

[1]Department of Physical Science, College of Science and Engineering, Ritsumeikan University, 1-1-1 Nojihigashi, Kusatsu, Shiga 525-8577, Japan.
[2]Disaster Prevention Research Institute, Kyoto University, Gokasho, Uji, Kyoto 611-0011, Japan.
[*]s-hrn@fc.ritsumei.ac.jp

ABSTRACT

In this supporting information, we provide some mathematical derivations for the Theory and Method section of the main text and a supplement table and figures. Notation and abbreviations are introduced in the main text.

Supplement A Approximation of the CC distribution

Here, we show that the normal distribution $N\left(0,d^{-1}\right)$ approximates the theoretical distribution of CC between two d-dimensional vectors $\boldsymbol{u}$ and $\boldsymbol{v}$ extracted from a continuous waveform record. First, we show that an extracted vector is statistically isotropic. From the definition, we consider $\boldsymbol{v}(t) = (x_t, x_{t+1}, \ldots, x_{t+d-1})$, where x_t is the t-th component of the continuous record. Therefore, $CC_t = \widehat{\boldsymbol{u}} \cdot \widehat{\boldsymbol{v}}(t)$ is the t-th value of CC if $\boldsymbol{u}$ is the fixed template, where $\widehat{\boldsymbol{u}}$ and $\widehat{\boldsymbol{v}}$ are normalized $\boldsymbol{u}$ and $\boldsymbol{v}$, respectively, after the elimination of their offset. If $t_0 \in [t, t+d-1]$ exists such that $|x_{t_0}|$ is significantly larger (or smaller) than others, $\widehat{\boldsymbol{v}}(t)$ itself is strongly (or less) oriented towards the t_0-th direction. However, simultaneously, $\widehat{\boldsymbol{v}}(t+1)$, $\widehat{\boldsymbol{v}}(t+2)$, $\widehat{\boldsymbol{v}}(t+3)\ldots$ are strongly (or less) oriented towards the t_0-1, t_0-2, $t_0-3\ldots$ directions; this discussion is obviously valid even if the continuous record has some coherence. Therefore, it is impossible to give some tendency to the direction of $\boldsymbol{v}$, that is, $\boldsymbol{v}(t)$ for all t is statistically isotropic.

Given the isotropy, the normal distribution $N\left(0,d^{-1}\right)$ can be obtained as the extension of the derivation of the Maxwell-Boltzmann distribution. However, Maxwell[1] assumed that each component of the vector, which is 3-dimensional and d-dimensional in the original and our problems, respectively, is independent; in our problem, this assumption does not hold because of $|\widehat{\boldsymbol{u}}|^2 = |\widehat{\boldsymbol{v}}|^2 = 1$. Therefore, we loosen this constraint as $E\left(|\widehat{\boldsymbol{v}}|^2\right) = 1$, where $E(\,\cdot\,)$ indicates the mean value. After the derivation, we justify this assumption for a larger value of d.

The derivation of the Maxwell-Boltzmann distribution is purely mathematical rather than physical. Maxwell[1] considered that each component of particle velocity $\boldsymbol{v} = (v_1, \ldots, v_d)$ is a random variable that follows the same PDF, P. Although only $d = 3$ was considered in the original, we extend it to the general case. In the following, we consider the PDF for each component of $\widehat{\boldsymbol{v}} = (\widehat{v}_1, \ldots, \widehat{v}_d)$.

By assuming that the random vector $\boldsymbol{v}$ is statistically isotropic (i.e., "*the directions of the coordinates are perfectly arbitrary*", Maxwell wrote), $E(\widehat{v}_j) = 0$ holds for arbitrary direction, and the joint probability of $\widehat{v}_1, \ldots, \widehat{v}_d$ is coordinate-free and depends only on $|\widehat{\boldsymbol{v}}|^2$ written as

$$\prod_{j=1}^{d} P\left(\widehat{v}_j\right) = \phi\left(\sum_{j=1}^{d} \widehat{v}_j^2\right). \tag{S.1}$$

Because an exponential function satisfies this property,

$$P\left(\widehat{v}_j\right) = \frac{1}{\alpha\sqrt{\pi}} \exp\left(-\frac{\widehat{v}_j^2}{\alpha^2}\right) \tag{S.2}$$

is obtained under the condition of $\int_{\mathbb{R}} P(x)dx = 1$, where α is a positive parameter to be determined. The joint probability is written as

$$\prod_{j=1}^{d} P(\widehat{v}_j) = \frac{1}{\alpha^d \pi^{d/2}} \exp\left(-\frac{|\widehat{\boldsymbol{v}}|^2}{\alpha^2}\right), \tag{S.3}$$

and the mean value of $|\widehat{\boldsymbol{v}}|^2$ is

$$\begin{aligned} E\left(|\widehat{\boldsymbol{v}}|^2\right) =& \int_{\mathbb{R}^d} |\widehat{\boldsymbol{v}}|^2 \prod_{j=1}^{d} P(\widehat{v}_j)\, d\widehat{v}_j && \text{(S.4)} \\ =& \frac{1}{\alpha^d \pi^{d/2}} \int_{S_{d-1}} d\omega \int_0^\infty |\widehat{\boldsymbol{v}}|^2 |\widehat{\boldsymbol{v}}|^{d-1} \exp\left(-\frac{|\widehat{\boldsymbol{v}}|}{\alpha^2}\right) d|\widehat{\boldsymbol{v}}| && \text{(S.5)} \\ =& \frac{2\pi^{d/2}}{\alpha^d \pi^{d/2} \Gamma(d/2)} \frac{1}{2} \alpha^{d+2} \Gamma\left(\frac{d}{2}+1\right) && \text{(S.6)} \\ =& \frac{\alpha^2 d}{2}, && \text{(S.7)} \end{aligned}$$

where $S_{d-1} = 2\pi^{d/2}/\Gamma(d/2)$ is the area of the $(d-1)$-dimensional unit sphere, $d\omega$ is the solid angle, and Γ is the Gamma function. $|\widehat{\boldsymbol{v}}|^{d-1}$ is derived from the Jacobian, and we use

$$\int_0^\infty x^p \exp\left(-\frac{x^2}{a^2}\right) dx = \frac{1}{2} a^{p+1} \Gamma\left(\frac{p+1}{2}\right). \tag{S.8}$$

Finally, with $E\left(|\widehat{\boldsymbol{v}}|^2\right) = 1$, we get

$$\alpha^2 = \frac{2}{d}, \tag{S.9}$$

which yields

$$P(\widehat{v}_j) = \sqrt{\frac{d}{2\pi}} \exp\left(-\frac{\widehat{v}_j^2}{2d^{-1}}\right). \tag{S.10}$$

Obviously, if d is small, eq.(S.10) does not approximate the distribution of CC because the probability is not negligible for $|\widehat{v}_j| > 1$. Hence, we have to consider the sufficiently large value of d that makes $P(|\widehat{v}_j| > 1)$ negligibly small. Moreover, the variance of $|\widehat{\boldsymbol{v}}|^2$ calculated as

$$\begin{aligned} E\left(\left(|\widehat{\boldsymbol{v}}|^2 - 1\right)^2\right) =& E\left(|\widehat{\boldsymbol{v}}|^4\right) - 2E\left(|\widehat{\boldsymbol{v}}|^2\right) + E(1) && \text{(S.11)} \\ =& \alpha^4 \left(\frac{d}{2}+1\right) \frac{d}{2} - 2 + 1 && \text{(S.12)} \\ =& \frac{2}{d}, && \text{(S.13)} \end{aligned}$$

means that the possibility of $|\widehat{\boldsymbol{v}}|^2 = 1$ in the strict sense becomes larger as d increases. Therefore, the constraint $|\widehat{\boldsymbol{v}}|^2 = 1$ is approximately satisfied for larger values of d.

Considering that both Maxwell's particle and our unit random vector are isotropic, the PDF (S.10) provides not only the specific component $\widehat{v}_j$ but also a component along all directions, including $\widehat{\boldsymbol{u}}$ in the same manner. Therefore, the inner product of an arbitrary random unit vector $\widehat{\boldsymbol{v}}$ extracted from a random continuous waveform and arbitrary fixed unit vector $\widehat{\boldsymbol{u}}$ approximately follows the normal distribution with the variance of d^{-1}.

Supplement B MLE of GEV parameters

MLE of GEV parameters is equivalent to solving the equations below[2,3] with respect to μ', σ', and k:

$$\begin{aligned} \sum_{i=1}^{N} \frac{z_i}{y_i} &= 0, \\ -N + \sum_{i=1}^{N} \frac{z_i}{y_i} \left(\frac{x_i - \mu'}{\sigma'} \right) &= 0, \\ \sum_{i=1}^{N} \left(z_i \log(y_i) + \frac{z_i}{y_i} \left(\frac{x_i - \mu'}{\sigma'} \right) \right) &= 0, \end{aligned} \tag{S.14}$$

where $y_i := 1 + (k/\sigma')(x_i - \mu')$ and $z_i := 1 + k - y_i^{-1/k}$ (note: k is opposite in sign between references[2,3]), and we eliminate some unnecessary coefficients. To solve them using the Newton-Raphson method, the Hessian matrix that is the derivative of eqs.(S.14) with respect to the 3 parameters should be calculated. Although the representations of the derivatives are slightly complicated, we simply compute the matrix by automatic differentiation using a small complex variable[4]. The initial values for iteration are given by L-moments[5].

Unfortunately, the Newton-Raphson method sometimes fails during its iteration due to the following reason. During the MLE process, we have to calculate the log-likelihood $\log P_{\mathrm{GEV}}(x_j \mid \mu', \sigma', k)$ for all the samples x_j, where μ', σ', k are not necessarily the MLE of the parameters, which is attributable to the iteration. In the case of $k < 0$, as mentioned in the main text, the PDF, P_{GEV}, for $x > \mu - \sigma'/k$ is zero. Therefore, we may substitute zero into P_{GEV} if $x_j > \mu - \sigma'/k$ holds, and the iteration stops due to the numerical error ($\log 0 = -\infty$). In particular, this error tends to occur if the sample includes abnormally large outliers. Hence, the MLE of GEV parameters is technically difficult in our case, and we may require some improvement in the algorithm.

Because we particularly focus on the case of the Gumbel distribution, the equations for maximum likelihood estimators are represented explicitly by taking $k \to \infty$:

$$\begin{aligned} \sum_{i=1}^{N} \left(1 - \exp\left(-\frac{x_i - \mu'}{\sigma'} \right) \right) &= 0, \\ -N + \sum_{i=1}^{N} \left(1 - \exp\left(-\frac{x_i - \mu'}{\sigma'} \right) \right) \left(\frac{x_i - \mu'}{\sigma'} \right) &= 0. \end{aligned} \tag{S.15}$$

Supplement C Errors in the maximum likelihood estimation

In eq.(5) of the main text, we assume that the maximum likelihood parameters for all N, $N - s$, and $N - s - 1$ data do not vary significantly because $N \gg s$ holds. Even so, the effect of the small difference in the parameters on AIC_s appears to be unclear. In the calculation of the log-likelihood, $\sum_{j=s+1}^{N} \log P(x_j \mid \mu', \sigma')$, even a negligibly small difference could be stacked and possibly become a significant amount.

However, we can show that the stacked amount is still negligible. Let the parameters (μ', σ') and (μ'', σ'') be the MLE using all N data and $N - s$ data, respectively. Therefore, the error of AIC (i.e., error of the log-likelihood) using the former instead of the latter has the same order as the Kullback–Leibler divergence

$$D(P_G(\mu'', \sigma''), P_G(\mu', \sigma')) = \int_{\mathbb{R}} P_G(x \mid \mu'', \sigma'') \log \frac{P_G(x \mid \mu'', \sigma'')}{P_G(x \mid \mu', \sigma')} dx. \tag{S.16}$$

This is equivalent to the loss function defined in eq.(3.1) of Akaike[6], and depends only on the second or higher order of $(\mu'' - \mu')$ and $(\sigma'' - \sigma')$ after the Taylor series expansion; see eq.(4.5) of Akaike[6]. Hence, any small error of $(\mu'' - \mu')$ or $(\sigma'' - \sigma')$ does not vary the log-likelihood significantly.

Another concern is the magnitude of the estimation errors of the parameters. We executed bootstrapping to estimate the errors for μ' and σ' in the case of No.01. The data are the maximum CC values from every 1

minute interval during the two years ($N = 2 \times 365 \times 24 \times 60 \sim 10^6$), where the CC values are summed up over 12 stations (i.e., $-12 < CC < +12$). After 1,000 iterations, we obtained their mean $\pm$ standard deviation as $\mu' = 0.936457 \pm 1.27 \times 10^{-4}$ and $\sigma' = 0.123199 \pm 9.93 \times 10^{-5}$. Therefore, their estimation errors are up to 0.1%, a negligible perturbation as seen in Figure S.5. Moreover, we applied the bootstrapping test to every interval of 61-day data (2009-07-01 to 2009-08-30, 2009-08-31 to 2009-10-30, 2009-10-31 to 2009-12-30, 2009-12-31 to 2010-03-01, 2010-03-02 to 2010-05-01, and 2010-05-02 to 2010-07-01) to check seasonal variation of μ' and σ' during a year. As a result, the variation was up to 3.0% in μ' (2009-10-31 to 2009-12-30 vs 2010-03-02 to 2010-05-01) and 4.4% in σ' (2009-10-31 to 2009-12-30 vs 2009-12-31 to 2010-03-01). Even though these variations from the small data are larger than the estimation error from the whole data, they are still small, as the blue lines in Figure S.5 indicate.

References

1. Maxwell, J. C. V. illustrations of the dynamical theory of gases.—part i. on the motions and collisions of perfectly elastic spheres. *The London, Edinburgh, Dublin Philos. Mag. J. Sci.* **19**, 19–32, DOI: 10.1080/14786446008642818 (1860).
2. Martins, E. S. & Stedinger, J. R. Generalized maximum-likelihood generalized extreme-value quantile estimators for hydrologic data. *Water Resour. Res.* **36**, 737–744, DOI: 10.1029/1999wr900330 (2000).
3. Coles, S. *An introduction to statistical modeling of extreme values*. Springer Series in Statistics (Springer, London, England, 2001), 2001 edn.
4. Squire, W. & Trapp, G. Using complex variables to estimate derivatives of real functions. *SIAM Rev.* **40**, 110–112, DOI: 10.1137/s003614459631241x (1998).
5. Hosking, J. R. M. L-moments: Analysis and estimation of distributions using linear combinations of order statistics. *J. Royal Stat. Soc. Ser. B (Methodological)* **52**, 105–124, DOI: 10.1111/j.2517-6161.1990.tb01775.x (1990).
6. Akaike, H. Information theory and an extension of the maximum likelihood principle. *2nd Int. Symp. on Inf. Theory.* 267–281 (1973).

Table S.1. Events from the JMA catalog, which caused the observed waveforms, used as the template. The mainshock is in the bottom.

No.	Origin Time (JST)	Lat.(°)	Long.(°)	Depth(km)	M
01	2011-06-29 19:32:38.80	36.190	137.953	4.2	3.4
02	2011-06-29 19:34:44.07	36.189	137.954	3.9	1.0
03	2011-06-29 19:34:48.99	36.188	137.952	3.3	1.5
04	2011-06-29 19:35:29.14	36.185	137.955	4.3	0.9
05	2011-06-29 19:36:58.98	36.192	137.952	4.5	0.1
06	2011-06-29 19:37:05.01	36.188	137.954	4.7	1.7
07	2011-06-29 20:03:21.31	36.195	137.947	3.8	-0.1
08	2011-06-29 20:04:07.47	36.193	137.953	4.6	2.8
09	2011-06-29 20:05:22.30	36.193	137.954	4.5	2.2
10	2011-06-29 20:17:50.64	36.193	137.953	4.2	1.8
11	2011-06-29 20:37:05.29	36.189	137.948	3.5	-0.1
12	2011-06-29 20:58:58.26	36.198	137.951	4.9	2.3
13	2011-06-29 21:08:53.36	36.189	137.955	3.6	0.8
14	2011-06-29 21:21:03.53	36.188	137.955	3.6	0.6
15	2011-06-29 22:02:11.19	36.187	137.958	4.1	0.5
16	2011-06-29 22:15:55.52	36.187	137.956	3.8	0.9
17	2011-06-29 22:17:59.47	36.186	137.953	3.6	-0.3
18	2011-06-29 22:23:20.40	36.187	137.954	3.2	1.3
19	2011-06-29 22:28:37.93	36.193	137.955	4.8	0.3
20	2011-06-30 00:11:40.79	36.189	137.956	5.4	0.2
21	2011-06-30 00:56:56.58	36.190	137.954	4.2	1.5
22	2011-06-30 01:26:44.45	36.194	137.953	4.0	0.5
23	2011-06-30 01:39:53.64	36.190	137.954	4.4	1.6
24	2011-06-30 04:45:28.48	36.194	137.952	4.6	2.4
25	2011-06-30 05:26:36.43	36.194	137.952	4.5	1.5
26	2011-06-30 07:53:02.28	36.188	137.952	4.6	0.3
27	2011-06-30 08:08:38.04	36.184	137.956	4.2	1.6
	2011-06-30 08:16:37.06	36.188	137.955	4.3	5.4

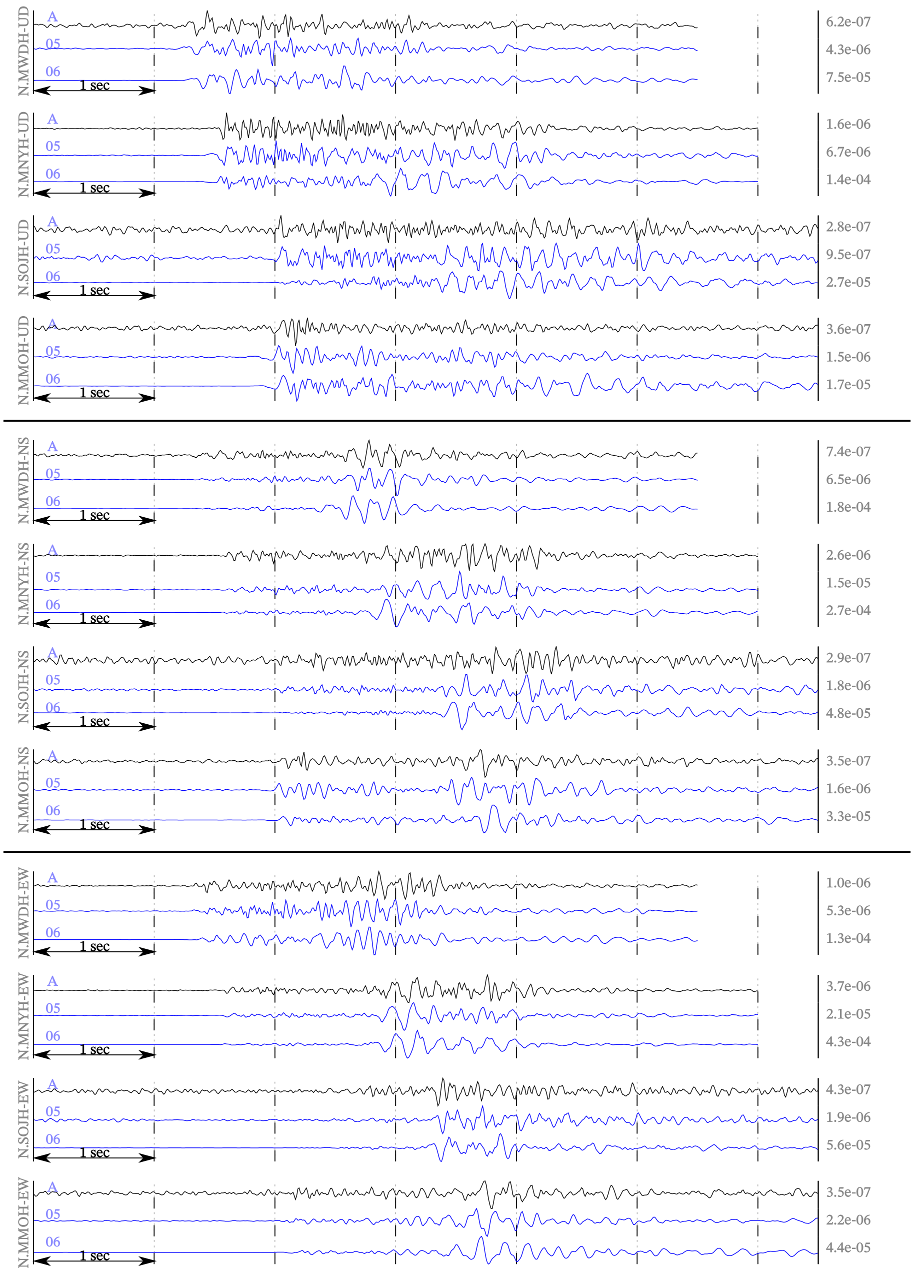

Figure S.1. UD, NS, and EW components of the detected (black) and template (blue) waveforms recorded at N.MWDH, N.MNYH, N.SOJH, and N.MMOH stations from top to bottom. The left edges of template waveforms are aligned at the origin time of each event. ID (A) and No. (05, 06) refer to Table 1 and S.1, respectively. The differences between the maximum and minimum values of each [m/s] are shown in the right side.

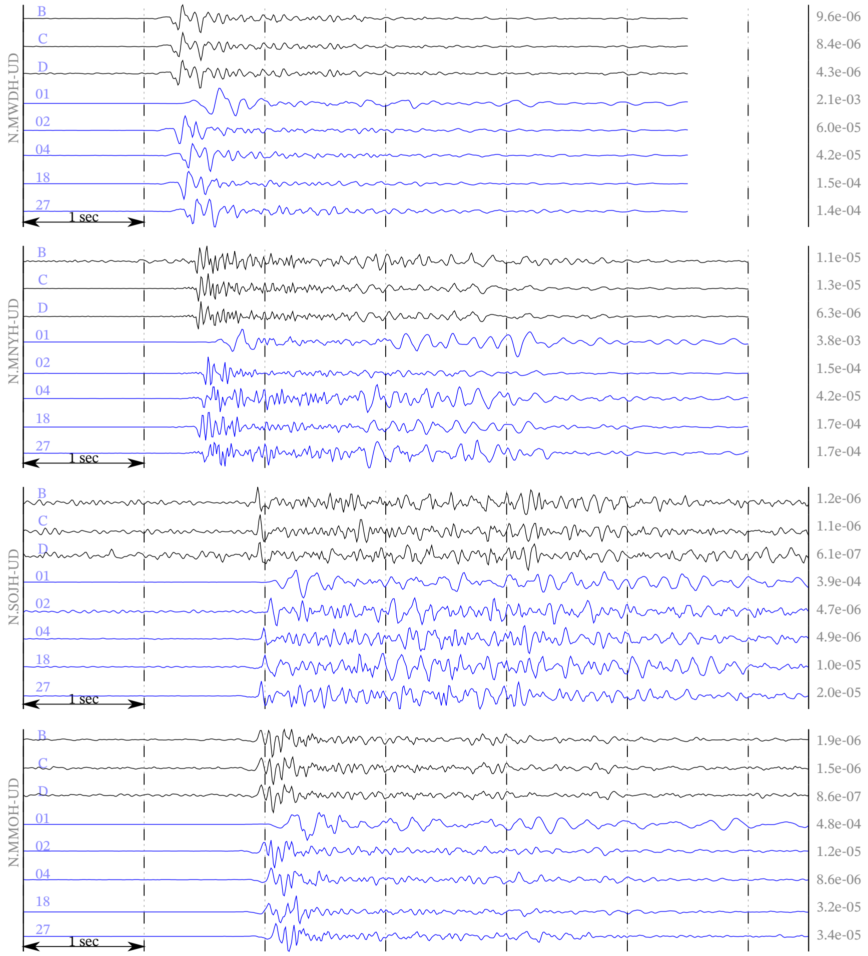

Figure S.2. UD component of the detected (black) and template (blue) waveforms recorded at N.MWDH, N.MNYH, N.SOJH, and N.MMOH stations from top to bottom. The left edges of template waveforms are aligned at the origin time of each event. ID (B, C, and D) and No. (01, . . . , 27) refer to Table 1 and S.1, respectively. The differences between the maximum and minimum values of each [m/s] are shown in the right side.

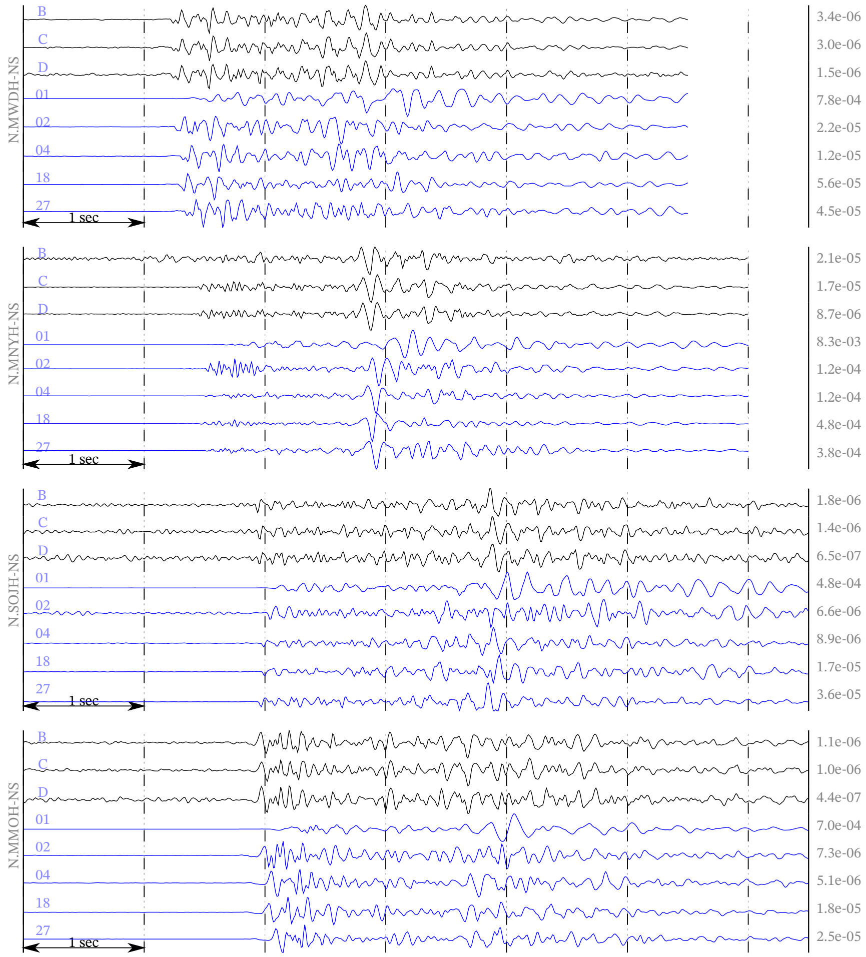

Figure S.3. NS component of the detected and template waveforms.

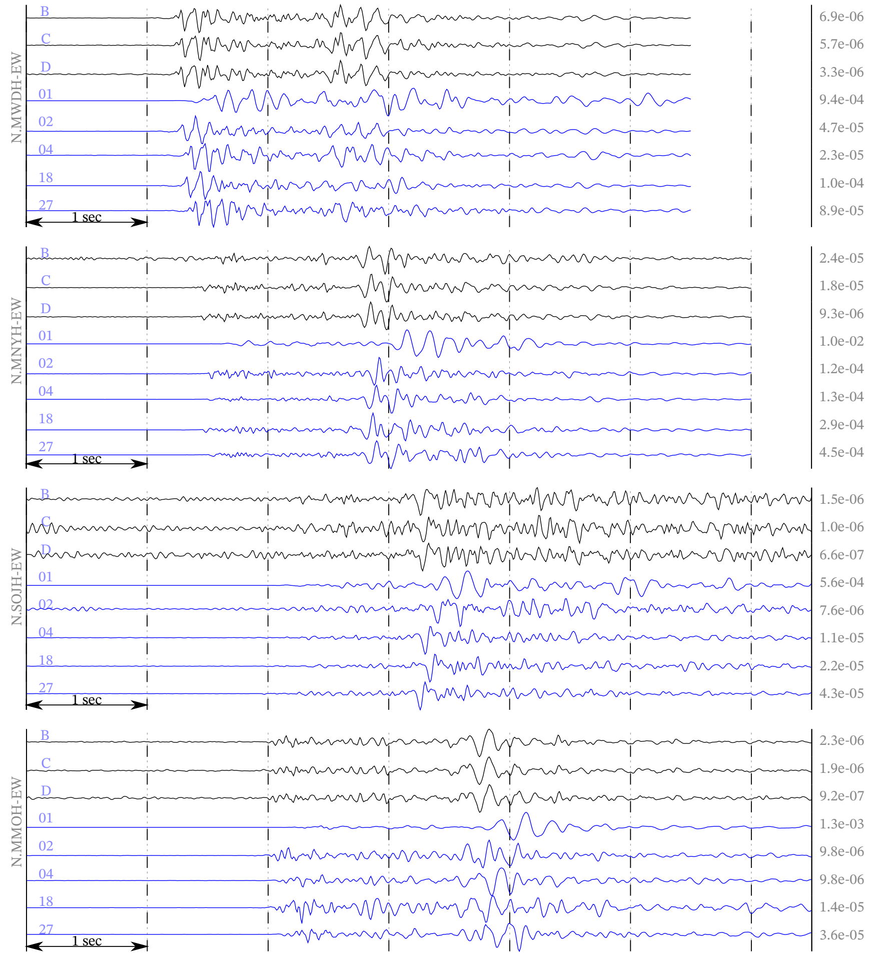

Figure S.4. EW component of the detected and template waveforms.

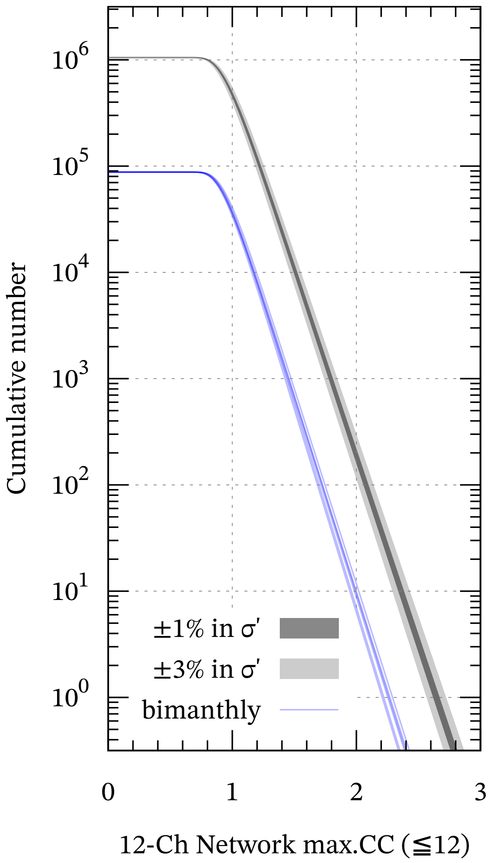

Figure S.5. Perturbation of the Gumbel distribution estimated based on the 2-year data with $\pm 1\%$ (dark gray) and $\pm 3\%$ (light gray) error in the estimation of σ'. Note that the estimated error after the bootstrapping is up to 0.1%, which is negligibly small in this figure. The blue lines indicate the distributions based on every interval of 61-day data to check the seasonal variation.
